# Role of Aging and Hippocampus in Time-Place Learning: Link to Episodic-Like Memory?

**DOI:** 10.3389/fnbeh.2015.00362

**Published:** 2016-01-19

**Authors:** C. K. Mulder, M. P. Gerkema, E. A. Van der Zee

**Affiliations:** ^1^Department of Molecular Neurobiology, University of GroningenGroningen, Netherlands; ^2^Department of Chronobiology, University of GroningenGroningen, Netherlands

**Keywords:** learning, circadian, memory, aging, time, place, cry, clock genes

## Abstract

**Introduction:** With time-place learning (TPL), animals link an event with the spatial location and the time of day (TOD). The what–where–when TPL components make the task putatively episodic-like in nature. Animals use an internal sense of time to master TPL, which is circadian system based. Finding indications for a role of the hippocampus and (early) aging-sensitivity in TPL would strengthen the episodic-like memory nature of the paradigm.

**Methods:** Previously, we used C57Bl/6 mice for our TPL research. Here, we used CD1 mice which are less hippocampal-driven and age faster compared to C57Bl/6 mice. To demonstrate the low degree of hippocampal-driven performance in CD1 mice, a cross maze was used. The spontaneous alternation test was used to score spatial working memory in CD1 mice at four different age categories (young (3–6 months), middle-aged (7–11 months), aged (12–18 months) and old (>19 months). TPL performance of middle-aged and aged CD1 mice was tested in a setup with either two or three time points per day (2-arm or 3-arm TPL task). Immunostainings were applied on brains of young and middle-aged C57Bl/6 mice that had successfully mastered the 3-arm TPL task.

**Results:** In contrast to C57Bl/6 mice, middle-aged and aged CD1 mice were less hippocampus-driven and failed to master the 3-arm TPL task. They could, however, master the 2-arm TPL task primarily via an ordinal (non-circadian) timing system. c-Fos, CRY2, vasopressin (AVP), and phosphorylated cAMP response element-binding protein (pCREB) were investigated. We found no differences at the level of the suprachiasmatic nucleus (SCN; circadian master clock), whereas CRY2 expression was increased in the hippocampal dentate gyrus (DG). The most pronounced difference between TPL trained and control mice was found in c-Fos expression in the paraventricular thalamic nucleus, a circadian system relay station.

**Conclusions:** These results further indicate a key role of CRY proteins in TPL and confirm the limited role of the SCN in TPL. Based on the poor TPL performance of CD1 mice, the results suggest age-sensitivity and hippocampal involvement in TPL. We suspect that TPL reflects an episodic-like memory task, but due to its functional nature, also entail the translation of experienced episodes into semantic rules acquired by training.

## Introduction

Natural environments show many daily dynamics. The availability of food, mates and predators varies across both space and time. If these stimuli vary predictably, it is advantageous for animals to learn this spatiotemporal day-to-day variability. The ability to encode spatiotemporal reoccurring events, and to exploit this information by efficiently organized daily activities, is believed to constitute a significant fitness advantage which has likely shaped the architecture of cognitive and circadian systems over the course of evolution. Indeed, the ability to learn spatiotemporal variability has been demonstrated in many species and has become known as time-place learning (TPL; for a review, see Mulder et al., 2013a). In TPL, animals have to link a stimulus with the location and the time of day (TOD). In our TPL paradigm (Van der Zee et al., 2008; Mulder et al., 2013a), food deprived mice are confronted with a conflict between a positive reinforcer (food reward) and a negative reinforcer (mild footshock) in a three-arm maze depending on the TOD. The paradigm emulates the natural situation in which hungry animals seek food while different feeding locations (arms of the maze) can be predictably safe or unsafe to visit in a TOD-dependent manner (Van der Zee et al., 2008). Investigating TPL can help to gain better understanding of the foraging dynamics in prey or predators. Besides this ecological relevance, TPL is interesting in the field of Neuroscience. Animals can use their circadian system for TPL, referred to as circadian TPL (cTPL). cTPL depends on *Cry*, but not *Per* clock genes (Van der Zee et al., 2008; Mulder et al., 2013b). However, insights into the specific role of the circadian system in memory formation is limited (Van der Zee et al., 2009; Mulder et al., 2013a; Smarr et al., 2014; and references therein). Moreover, cTPL does not depend on the circadian master clock, the suprachiasmatic nucleus (SCN) or rhythmic release of corticosteron from the adrenals (Mulder et al., 2014). Therefore, cTPL must be primarily driven by non-SCN oscillators such as hippocampal cell assemblies, although the SCN may still play a modulatory role.

The components what–where–when in TPL make the task putatively episodic-like in nature. The “what” component is the food reward vs. the mild footshock at the food location, the “where” is one of the three arms, and the “when” is the TOD, predicting which arm can be visited for a food reward without getting the mild but aversive footshock. cTPL implies that distinct phases of an internal circadian clock can be incorporated in associative “what–where–when” memory. This type of memory is particularly susceptible to perturbations of aging and neurodegenerative diseases affecting the hippocampus, yet animal models to study episodic memory or episodic-like memory are scarce (Binder et al., 2015 and references therein).

Two typical aspects of episodic memory are the hippocampus-dependent and aging-sensitive nature of the task. To further explore to what extent cTPL is aging-sensitive, our first aim is to examine the CD1 mouse in a variety of behavioral studies including TPL. CD1 mice differ in the degree of hippocampal contribution to learning and memory performance as compared to C57Bl6 mice. The CD1 (albino) mouse is one of the most commonly used outbred stock, also in spatial learning tasks (see Patil et al., 2012, and references therein). The mice of this strain age relatively fast compared to C57Bl/6 mice (Chia et al., 2005; Johnson, 2014). Studies in mice (Havekes et al., 2011), rats (Begega et al., 2001), and humans (Yamamoto and Degirolamo, 2012) show that aged individuals tend to switch from a hippocampal-driven strategy to a striatal—driven strategy to solve spatial tasks, mainly because of the gradual loss of hippocampal function and aging-related loss of hippocampal neurogenesis and spine-plasticity needed for memory formation (Kuhn et al., 1996; Gil-Mohapel et al., 2013; Van der Zee, 2015). Adult hippocampal neurogenesis is required for spatial learning, since inhibition of hippocampal neurogenesis can impair spatial relational memory (whereas boosting adult neurogenesis by way of running wheel activity strongly improves hippocampus-dependent learning; van Praag et al., 2005; Van der Borght et al., 2007). Higher proliferation and higher neuronal survival has been shown in the hippocampus of C57Bl/6 mice compared to CD1 mice. On the other hand, higher net neurogenesis, higher volume of the DG and more granule cells have been found in CD1 mice (Kempermann et al., 1997; Gage, 2002), possibly to compensate for reduced hippocampal functioning. Moreover, equal environmental enrichment is more effective in stimulating hippocampal neurogenesis in C57Bl/6 mice than CD1 mice (Gage, 2002), suggesting a less flexible hippocampal system in the latter strain. It is not known how these differences in hippocampal neurogenesis and the higher rate of aging relate to TPL performance in CD1 mice. Therefore, the (aged) CD1 mouse seems a suitable strain to test whether mastering the TPL task is sensitive to relative poor hippocampal functioning.

As cTPL presumes a functional connection between the circadian system and memory system(s), our second aim is to explore putative neurobiological correlates of cTPL. This was done using immunohistochemistry (IHC) in brain sections from young and middle-aged C57Bl/6 mice that had successfully mastered the cTPL task. The markers we chose were c-Fos, CRY2, AVP, and pCREB. C-Fos belongs to the immediate early gene (IEG) family of transcription factors. Because IEGs are rapidly induced by neuronal activity, c-Fos is widely used as a marker for activated circuits at cellular scale (Morgan et al., 1987; Sagar et al., 1988; Kawashima et al., 2014). CRY2 is the transcription product of the core molecular clock gene Cry2 (Cryptochrome 2). The Cry genes are specifically interesting to investigate as neuronal markers for TPL, as TPL depends on Cry1 and/or Cry2 (Van der Zee et al., 2008), but not Per1 and Per2 clock genes (Mulder et al., 2013b). AVP (arginine vasopressin) is seen as the major output signal of the SCN master clock (Kalsbeek et al., 2010). Approximately 10–30% of the neurons within the SCN contain AVP. Vasopressin is indicated as the (humoral) output of the SCN because AVP producing SCN neurons project to distal targets areas, such as to the paraventricular nucleus (PVT). But AVP also has excitatory action within the SCN acting on the V1-type receptors (Kalamatianos et al., 2004). Salient events have been shown to induce a circadian rhythm in the expression of muscarinic acetylcholine receptors in the SCN, with peak expression levels coinciding with the event-specific TOD (Van der Zee et al., 2004). It has therefore been proposed that the SCN may function as a programmable “alarm clock”, using the neuropeptide AVP as an output to transfer the specific TOD information to other brain regions (Biemans et al., 2003; Van der Zee et al., 2004; van der Veen et al., 2008; Hut and Van der Zee, 2011). pCREB is a widely used marker for neuronal plasticity. CREB (cAMP response element-binding protein) is a cellular transcription factor which binds to certain DNA sequences called cAMP response elements (CRE), thereby increasing or decreasing the transcription of downstream genes. The phosphorylated form of CREB (pCREB) has been shown to be integral in the formation of spatial memory. Moreover, pCREB has a well-documented role in neuronal plasticity and protein synthesis-dependent long-term memory formation in diverse behavioral paradigms among many species (Bernabeu et al., 1997; Guzowski and McGaugh, 1997; Lamprecht et al., 1997; Colombo et al., 2003; Countryman et al., 2005). pCREB stimulates the expression of several IEGs. One of those genes is the proto-oncogene transcription factor c-Fos (Sheng and Greenberg, 1990). These four markers could provide a first glance of the neuronal substrate underlying cTPL.

Taken together, the current experimental data may shed more light on: (a) the age-sensitivity of cTPL in a strain-dependent manner and (b) the neuronal substrate underlying TPL. These findings may help to determine to what extent TPL can be viewed as an episodic-like memory task.

## Materials and Methods

### Animals and Housing

Male mice were housed individually in macrolon type II cages (length 35 cm, width 15 cm, height 13.5 cm, Bayer, Germany), with sawdust as bedding and shredded cardboard as nesting material. The mice were kept in a climate room with controlled temperature (22 ± 1°C) and humidity (55 ± 10%). A light/dark (LD) schedule (12 h light-12 h dark; lights on at 07:00 h GMT+1 h) was maintained. Light intensity was always 20–50 lux measured between the cages. Food (standard rodent chow: RMHB/2180, Arie Block BV, Woerden, Netherlands) was available *ad libitum*, except during food deprivation (cross maze and TPL testing). Normal tap water was available *ad libitum*. Cages were cleaned at least once every 2 weeks. All mice were checked daily for food/water/health/activity/abnormal behavior. The protocol was approved by the ethical committee for the use of experimental animals of the University of Groningen. All efforts were made to minimize the number of animals used and their discomfort. For this study four age categories are defined as follows: young (3–6 months), middle-aged (7–11 months), aged (12–18 months), and old (>19 months). An overview of the used mice and the age categories is provided in Table 1.

**Table 1:** **Overview of the mice used in the experiments**.

Experiment	CD1		C57Bl/6		Remarks
	*N*	Age	*N*	Age
cross maze	9	3–6 mo (Y)	15	3–6 mo (Y)
	9	>19 mo (O)	8	>19 mo (O)
SA and circadian score	8	3–6 mo (Y)
	7*	7–11 mo (MA)
	7*	12–18 mo (A)
	4	>19 mo (O)
3-arm TPL and 2-arm TPL	9	7–11 mo (MA)			3-arm TPL design
	7*	7–11 mo (MA)			2-arm TPL-design
	7*	12–18 mo (A)			2-arm TPL-design
3-arm TPL and Immunostaining (IHC)			7 + 8 HCC	3–6 mo (Y)	CRY2, AVP, c-Fos,
					pCREB
			9 + 5 HCC	7–11 mo (MA)

Overview of the mice used in the cross maze, the spontaneous alternation test (SA), the circadian score experiment, and the 3-arm and 2-arm TPL test. For the immunostaining experiment, the age indicates the age at which the mice were sacrificed. CD1 mouse numbers indicated with an asterisk (*) are the same mice per age category used in the different tests. HCC: Home Cage Controls. IHC: Immunohistochemistry. Age categories are indicated between parentheses [Y: Young (3–6 months); MA: Middle-Aged (7–11 months); A: Aged (12–18 months); O: Old (>19 months)].

### Cross Maze Test

To test for strain-dependent preferences for a hippocampal-driven strategy in place learning, a cross maze consisting of a small chamber with transparent ceiling and four arms (tubes) arising from it was used. For each mouse, the following protocol was used: 5 days before the beginning of the experiment mice were food deprived. Testing started when the body weight of the mice was about 85% of their body weight at the start of the experiment. The experiment was divided into three phases: the habituation phase lasted 1 day on which each mouse performed two trials with a 3 min interval (during which mice remained in their home cage in the experimental room). The mouse was put in a transporter cage which was then connected to the maze. It entered the maze from the south arm, while the north arm was closed. During the first trial, four small pieces of food were present (standard rodent chow; one piece of food was approximately the size of a pencil tip), two in the beginning of the right and left arm and two at the end of those arms. During the second trial, only the end of the right and left arms were baited with food (one piece of food per arm). When the mouse entered the maze, the way back to the transporter cage was blocked. The mouse was allowed to exit the maze from the same arm it got in after it had consumed all the food, or when 5 min had passed. After each trial the maze was cleaned with 30% ethanol and towel-dried. The next phase was the training phase. Each mouse performed six trials per day with a 3 min interval after each trial. Mice entered again from the south arm, while the north arm was closed. It this phase, only one arm was baited with food (randomized between mice). Under the perforated non-baited arm, a small piece of food was present (not visible and out of reach of the mouse) to rule out the possibility that mice selected the correct arm by olfactory cues. Mice had to learn to visit the baited arm first in order to get to the food. The non-baited arm was then blocked and the mouse was allowed to exit the maze. A trial was considered successful if the mouse entered the baited arm first. The training phase ended when a mouse performed five correct trials in a row on a single day. The last phase was the testing phase in which the mouse performed only one trial. This time they entered the maze from the north arm, while the south arm was blocked. Both right and left arms were baited with food. If the mouse visited the trained arm first, it indicated a preference for a hippocampal-driven strategy.

### Running Wheel Activity and Circadian Score

To measure internal clock stability, animals were housed in cages equipped with a running wheel (diameter 13.5 cm) for constant activity recording. An 11 day constant dim light (LL) period was introduced, during which the animals display their endogenous activity rhythm (free-run). For each mouse, daily activity onsets during this period were determined by using a high- and low pass filter (crossing of 24 h-, and 4 h running-means). A linear trend line was calculated through these onset data points and absolute distances from each onset data point to this trend line were determined. A “circadian score” for a mouse was calculated (defined by: [average of absolute distances from trend line]^−1^ × 100%, creating a score ranging from 0–100%, higher values indicating more consistency in activity onsets under free-run conditions).

#### Spontaneous Alternation Test

This test (described before in more detail, see Mulder et al., 2013a) consisted of 8 min exploration trials in a three arm symmetrical maze and was based on the natural behavior of animals to explore locations that are novel or visited the longest time ago, relying on spatial working memory. An alternation was defined as a triplet of sequential unique location visits. The alternation score (SA score) was calculated by dividing the number of alternations by the total possible alternations, the latter being equal to the total number of entries minus two.

### TPL Testing Procedure

The used TPL test apparatus and testing procedures were described before (Figure 5A, 3-arm TPL design; Van der Zee et al., 2008; Mulder et al., 2013a,b, 2014, 2015). Briefly, to induce food seeking behavior and voluntary location-choices, mice were food deprived to 85% of their *ad libitum* body weight, as individually determined by the average of three daily measurements prior to initiating food deprivation. To monitor bodyweight during testing, mice were weighed before each daily TPL test session and received an individual amount of food at the end of the light-phase (ZT10.5).

After habituation steps (as described previously in Van der Zee et al., 2008; Mulder et al., 2013a), TPL testing (2-arm TPL design or 3-arm TPL design; see Figure 3) was commenced. In each of the daily test sessions (lasting maximally 10 min per mouse; two daily sessions in the 2-arm TPL design and three daily sessions in the 3-arm TPL design; see Figure 3), mice were presented with two or three different feeding locations. The mice had to learn to avoid one “non-target” location, which changed depending on the TOD (i.e., session). As a punishment for visiting the non-target location, mice received a mild but aversive footshock (set to 620 volts; 0.09 mA; <1 s). All locations were baited with powdered standard rodent chow (<0.1g) so that mice could not identify the non-target/target location(s) based on sight/smell and had to use knowledge of circadian phase to discriminate the hazardous non-target location. A session was considered correct, on an individual level, only when the two target locations were visited first, avoiding the non-target location or visiting it lastly. Daily performance was calculated for each animal as the percentage of correct sessions and these performances were averaged, forming a learning curve over multiple testing days (Figure 5B). Mice were tested in their inactive (light-) phase. A session (session 1 in the 2-arm TPL design and session 1 or 2 in the 3-arm TPL design) can be skipped to determine whether the mouse mastered the task using a circadian or a ordinal (non-circadian) strategy (Figure 4). Performance should drop below chance level if mice use an ordinal (non-circadian) strategy, which is presumably less hippocampus dependent. Home cage control (HCC) mice were not TPL tested, but similarly food deprived. In the 2-arm TPL test, middle-aged and aged mice were alternated in order of testing, so that a potential “time of day effect” was equally divided over the two groups. The location of the shock-arm was also alternated between trials to exclude the possibility that mice follow potential scent trails of the previously tested animal. The maze was cleaned between trials with a wet paper cloth.

### Collection and Processing of Brain Material

Mice (in couples of a HCC and TPL mouse) were sacrificed at the time of their first or second daily TPL test-session (deviation maximally 10 min). We selected these time points for practical reasons, but avoided selecting the third TPL session time point because expression of most clock genes is lower at the beginning of the light-phase. Hence, by choosing the earlier time points we increased the detectability of potentially upregulated markers (compared to HCC mice) in anticipation of TPL testing.

Under deep pentobarbital anesthesia, mice were perfused transcardially for 1 min with 0.9% NaCl + 0.5% heparin (400U) in H_2_O (15 ml/min), followed by 150 ml 4% paraformaldehyde (PF) in 0.1 M phosphate buffer (PB) for fixation. Brains were collected and further processed in Greiner cups (Greiner Bio-One, Container, PS, 15 ml, 40 × 24.5 mm snapdeks, cat #203170). Brains were postfixated for 24 h in 4% PF in 0.1 M PB, rinsed for 1 day in 0.01M phosphate buffered saline (PBS, pH 7.4) and then kept overnight in 30% sucrose in PBS cryoprotectant at 4°C. Brains were frozen the next day using liquid nitrogen and stored at −80°C until further processing. Brains were cut in coronal sections of 25 μm thick using a cryotome and stored at 4°C. Target areas were the SCN (these sections also containing the anterior PVT) which was cut from −0.34 to −0.70 relative to bregma, and the hippocampus (these sections also containing PVT and cortex) which was cut from −1.82 to −2.06 relative to bregma according to the mouse brain stereotaxic atlas (Franklin and Paxinos, 1997, Academic press, CA, USA). Sections were sequentially distributed over eight Greiner cups containing 0.01M PBS, to create multiple equal series that could be used for different immuno-stainings.

### Immunohistochemistry

Three to five brain sections per mouse were used for each staining. Because similar protocols were used for each staining, only the procedures for the pCREB staining will be described as an example. Brain sections were rinsed three times for 5 min in TBS (0.01 M Tris-HCL + 0.9% NaCl, pH = 7.4), and were then placed in 0.3% H_2_O_2_ in TBS for 30 min. After rinsing the sections in TBS four times, 5 min each time, the primary antibody solution was added (Rabbit α-pCREB Millipore 1:1000 with 5% Normal Goat Serum and 0.1% Triton-X 100 in TBS). Sections were incubated overnight at room temperature on a shaker. After being rinsed with TBS eight times, for 10 min each time, the sections were incubated at room temperature for 2 h with the secondary antibody (biotinylated Goat anti-rabbit IgG Jackson 1:500 with 1% Normal Goat Serum and 0.1% Triton-X 100 in TBS). Next, sections were rinsed eight times with TBS for 10 min each time. After that, the sections were put in ABC complex (1:500 in TBS) for 2 h and then rinsed again eight times with TBS for 10 min each time. Finally, the labeled cells were visualized with diaminobenzidine (DAB, 0.7 mg/mL in H_2_O; Sigma-Aldrich, Steinheim, Germany) with 0.1% H_2_O_2_ as a reaction initiator. The reaction was stopped by rinsing three times with TBS for 5 min each time, and stored overnight in TBS at 4°C. The following day, the slices were mounted from a 1% gelatin in aquadest solution onto microscopic glasses using a paintbrush. The sections were placed with the posterior side faced up (during cutting the left hemisphere was marked so it could be distinguished during mounting) and ordered from anterior to posterior. Sections were left to dry overnight. Next, sections were put through an alchohol-xylol concentration series and covered with a cover glass using DPX mountant. A similar protocol was used for the other immunostainings, using different primary, and matching secondary antibodies. For CRY2, the primary antibody used was rabbit polyclonal anti-mCRY2 (1:200, from Alpha Diagnostic, USA). The available antibody for CRY1, the paralog of CRY2, failed to give a specific signal. For c-Fos, a rabbit polyclonal anti-c-Fos AB-5 was used (1:8000, vector), and for AVP a monoclonal anti-AVP (1:1000, PS41, kindly supplied by Dr. H. Gainer, NIH, MD, USA; Bult et al., 1992; Gerkema et al., 1994) was used.

### Quantification

For each staining, the most appropriate quantification method was determined. When only few specifically labeled cells were present in the area of interest or when a variable background was present, cells were manually counted through a microscope. This applies for CRY2 in the SCN and DG, and for c-Fos in the DG and PVT. For the other immunostainings, optical densities (OD) were measured at 50× magnification using a computerized image analysis system (Quantimet 550, Leica, Cambridge, UK). The OD is expressed in arbitrary units corresponding to gray levels. To correct for variability in background staining among sections, background labeling was measured in the corpus callosum and extracted from the OD of the area of interest. Bilateral measurements were averaged. The experimenter was blind to the treatment of individual animals during all cell counting and OD measurements. Because of the different quantification methods used, all results are expressed as percentage relative to the HCC group. Differences between TPL and HCC groups were tested by two-tailed unpaired *t*-tests using Microsoft Excel.

### Statistical Analysis

Statistical analyses were performed using GraphPad Prism 5.01 (GraphPad software, Inc.). Non-parametric tests were used in case datasets did not pass the normality test for Gaussian distribution (Kolmogorov-Smirnov). Differences in cross maze performance were tested using the Fisher Exact test. Differences between groups were analyzed using *t*-tests or the Kruskal-Wallis test with Dunn’s Multiple Comparison post-test. Differences from chance level were analyzed using one-sample *t*-test or the Wilcoxon Signed Rank Test. *P* < 0.05 was considered significant.

## Results

### Cross Maze, Spontaneous Alternation Task and Circadian Rhythmicity

**Figure 1:**
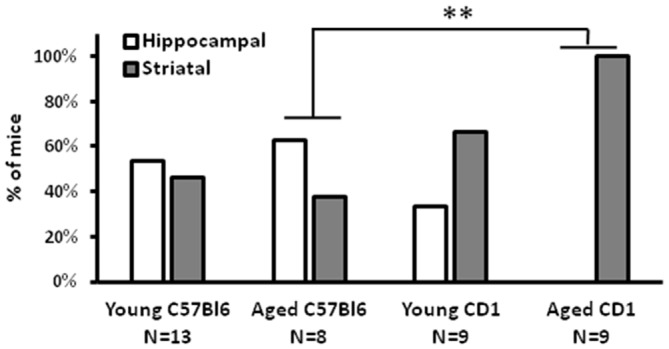
**Cross maze performance of CD1 mice.** A strong preference for a striatal-driven strategy was found in aged CD1 mice, whereas in C57Bl/6 and young CD1 mice no clear preference for either a hippocampal- or striatal-driven strategy was present. **p < 0.01, Fisher exact test.

For an overview of the used mice (strain, number and age) and tasks see also Table 1. The cross maze task revealed that notably the old CD1 mice (*N* = 9; >19 months of age) are less hippocampal-driven than old C57Bl/6 mice (*N* = 8; >19 months of age) in a spatial learning task (Figure 1; Fisher exact test; *p* = 0.009). Out of the nine aged CD1 mice, none had a preference for a hippocampal-driven strategy. This clear bias towards a striatal-driven strategy indicates a reduced hippocampal functioning in the aged CD1 mice as compared to the aged C57Bl/6 mice. For young mice (*N* = 9 for both strains; 3–6 months of age), such a difference was not present, although a higher percentage of young CD1 mice (67%) had a preference for a striatal-driven strategy as compared to the young C57Bl/6 mice (46%). These results demonstrate CD1 mice to be less hippocampal-driven than C57Bl/6 mice at young and particularly old age.

**Figure 2:**
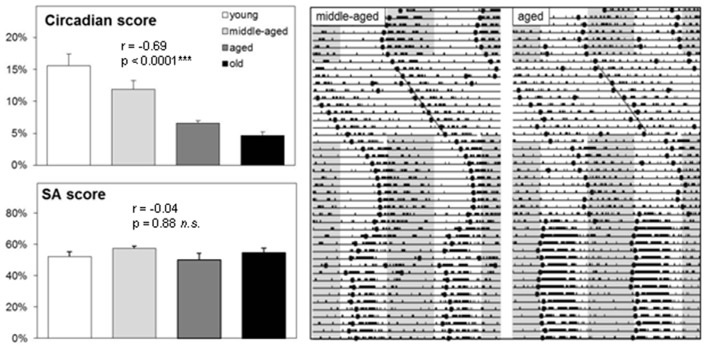
**Running wheel activity and spontaneous alternation (SA) performance of CD1 mice.** Left: Group averages of circadian score and SA score (see text for further details on the correlations). Error bars represent SEM. Right: Representative double-plotted actograms (46 days) of a middle-aged and aged CD1 mouse. Gray areas indicate darkness (12:12 LD). Mice were kept under a 12:12 light-dark cycle, except during a constant light (LL) period of 11 days to asses free-run patterns. The circadian score was based on the average distance of calculated daily activity onsets (black dots) during LL to a linear trend line.

Next, we determined the impact of aging in the Spontaneous Alternation (SA) paradigm and the circadian organization of behavior based on running wheel activity (Figure 2). Spatial orientation and stability of the circadian system were measured, together with TPL performance, in middle-aged (*N* = 7; 7–11 months of age) and aged (*N* = 7; 12–18 months of age) male CD1 mice. Figure 2 shows a representative actogram of a middle-aged and aged mouse, together with the calculated onset data points and trend line drawn through these. The upper left graph shows average circadian scores per age group. Two other groups of young (*N* = 8; 3–6 months of age) and old (*N* = 4; >19 months of age) male CD1 mice were included in this analysis. The circadian score declined with aging (Spearman *r* = − 0.69, *p* < 0.0001). The Kruskal-Wallis test showed that the age groups were significantly different (KW statistic = 18.81; *p* = 0.0003). Dunn’s post-test showed significant differences between young and aged (*p* < 0.05), young and old (*p* < 0.001), and middle-aged and old (*p* < 0.05) mice. Besides the circadian score extracted from the actograms, other signs of aging were also apparent, including increased fragmentation/noise and decreased general running wheel activity.

Performance in the SA paradigm was used as a measure of spatial working memory. Again, two additional age groups were tested to investigate more thoroughly the relation between aging on spatial working memory (young: *N* = 4; 3–6 months of age, old: *N* = 4; >19 months of age). The SA score stayed relatively robust with aging (Spearman *r* = − 0.04, *p* = 0.88). The Kruskal-Wallis test showed that the age groups did not differ significantly from each other (KW statistic = 3.80; *p* = 0.28). Dunn’s post-test showed no significant differences between age groups. Taken together, these results show that a decline of the strength in circadian organization of running wheel activity with aging in CD1 mice, indicating reduced functioning of the circadian system. In contrast, no such decline was found for SA performance. Of note, SA performance in C57Bl/6 mice in our hands is usually around 70% (Mulder et al., 2014), indicating that CD1 mice have a weaker SA performance than C57Bl/6 mice and hence a reduced hippocampal functioning as the hippocampus is a critical brain region for spatial working memory.

### Time Place Learning in CD1 Mice: 3-arm- and 2-arm Design

**Figure 3:**
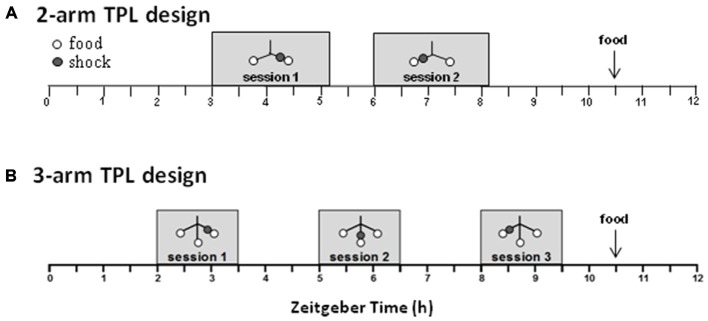
**Schematic overview of the daily time-place learning (TPL) testing protocol for two time points and two daily sessions (2-arm TPL design) or three time points and three daily sessions (3-arm TPL design).** Mice had to learn to avoid one non-target location, which changed depending on the time of day (TOD; i.e., session). Open circles indicate food (powdered standard rodent chow, <0.1 g) at the end of an arm of the maze (see Figure 5A); gray circles indicate the (non-target) shock location. Mice were tested individually two or three times a day (depending on the design) in 10 min trials, with an intersession time of 3 h. Bodyweights were taken before each trial. Mice received an individual amount of food at the end of each day in order to maintain body weight at 85–87% of *ad libitum* feeding weight. Testing was performed in the light phase. ZT0 (zeitgeber time zero) indicates lights on.

**Figure 4:**
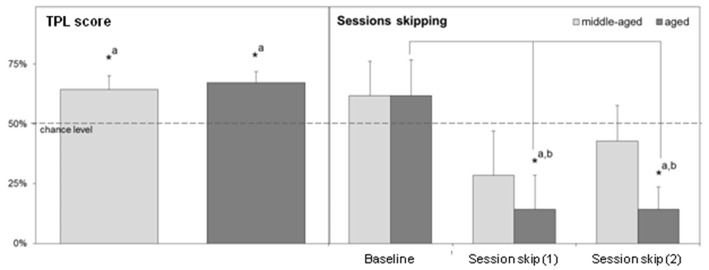
**Left:** Average TPL performance in the 2-arm TPL setup of middle-aged and aged CD1 mice over the last 10 days of testing. **Right:** Performance after two separate morning session (session 1) skips measured in the second session. For statistical comparison, a “baseline” of second session performance around the time of the session skips is also shown. Sessions were skipped on days 15 and 17. The baseline consists of average performances in session 2 on days 13, 14 and 16. Chance level is indicated by the horizontal dotted line in both figures. Error bars represent SEM. Significant differences (*p < 0.05) with chance level are indicated by superscript “a”; significant differences with baseline are indicated by superscript “b”.

**Figure 5:**
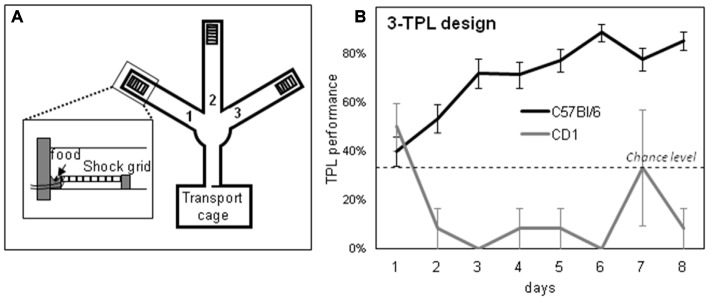
**A schematic representation of the 3-arm TPL test apparatus (A) and the learning curve of young C57Bl/6 mice (B; C57Bl/6 mice gradually mastered the task)**. Chance level is at 33%. The inset in **(A)** shows the shock grid and food location at the end of an arm. In a target arm, no footshock is delivered, whereas in a non-target arm food and footshock delivery are combined.

The designs of the 2-arm and 3-arm TPL tasks are depicted in Figure 3. A schematic representation of the maze including the shock device is given in Figure 5A. In case of the 2-TPL setup, the middle arm was blocked. The study with nine middle-aged CD1 mice revealed that these mice were unable to learn the 3-arm TPL setup, irrespective of age. None of the mice were able to perform above the 33% chance level. For this reason a simpler TPL design was used in which fourteen CD1 mice (seven middle-aged and seven aged, (Table 1)—these are the same mice as used for the SA and circadian scores described above) had to associate only two locations at two different time points (sessions), with a chance level of 50% correct scores. Average group performance on the first 4 days did not differ significantly from chance level for both middle-aged and aged mice (mean 55.6% ± *SD* 16.0, Wilcoxon Signed Rank Test *p* = 0.50 and mean 48.6% ± *SD* 18.3, Wilcoxon Signed Rank Test *p* = 0.78 respectively, data not shown). Average group performances over the remaining last ten testing days (5–14) are shown in Figure 4 (left panel). One sample *t*-test (two-tailed) showed that both middle-aged and aged mice performed significantly above chance level (*p* = 0.049 and *p* = 0.01, respectively). Results of middle-aged mice just reach significance, but mainly because of one low performing animal with an average performance of 40%. Unpaired *t*-test showed no significant difference between middle-aged and aged mice (*p* = 0.71, two-tailed).

Session skips reveal if animals use the skipped session as a cue in TPL and thus use an ordinal (non-circadian) strategy. When this is the case, performance should drop below chance level. The results of two separate morning session skips are shown on the right panel of Figure 4. Since the first session was skipped, performances represent daily group averages of only the second session. For comparison, a baseline for session 2 performance was determined based on 2 days before the first session skip and the 1 day between the first and second session skip.

For aged mice, performance after session skips was significantly different from baseline performance (Kruskal-Wallis test, *p* = 0.026). Dunn’s post-test showed that performance after both individual session skips was significantly different from baseline performance (*p* < 0.05). Moreover, after both session skips, performance of aged mice was significantly below chance level (Wilcoxon Signed Rank Test, one-tailed, *p* = 0.04 in both cases). Together this indicates that the aged mice use an ordinal (non-circadian) strategy for TPL.

Session skipping also decreased average performance of middle aged mice compared to baseline, but less and not significantly (Kruskal-Wallis test, *p* = 0.47). Dunn’s post-test showed that performance after both session skips was not significantly different from baseline performance (*p* > 0.05 in both cases). After both session skips, performance did not fall significantly below chance level (Wilcoxon Signed Rank Test, one-tailed, *p* = 0.15 and *p* = 0.39 for the first and second session skip respectively). Overall, middle-aged mice seemed less affected by both session skips, suggesting that these mice may also partly rely on another strategy besides ordinal. Middle-aged mice showed an increase in performance (but not significant) after the second session skip compared to the first session skip which may indicate an adaptation due to the first session skip during which these animals may have learned that the ordinal (non-circadian) strategy is no longer reliable. Aged mice did not show this kind of adaptation.

### Neuronal Substrate Candidates for (c)TPL

Two separate batches of C57Bl/6 mice were trained in the 3-arm TPL setup (Table 1). Young mice from the first batch were trained for 36 days, and middle-aged mice from the second batch were trained for 44 days. Learning curves from both batches were similar. The average learning curve is depicted in Figure 5B, which was comparable to previously published learning curves for the 3-arm TPL task (Van der Zee et al., 2008; Mulder et al., 2014). These mice were sacrificed the day after their last TPL test day, at the time of their first (batch 2) or second (batch 1) daily test session, together with HCC mice. All mice had been similarly food deprived (see “Materials and Methods” Section for more details). CD1 mice were not studied because (a) none of the CD1 mice mastered the 3-arm TPL task and (b) CD1 mice mastering the 2-arm TPL task did not use a circadian but instead an ordinal (non-circadian) strategy. Likewise aged C57Bl/6 mice are unable to master the 3-arm TPL task, unless they were trained in this task earlier in their life (Mulder et al., 2015).

**Figure 6:**
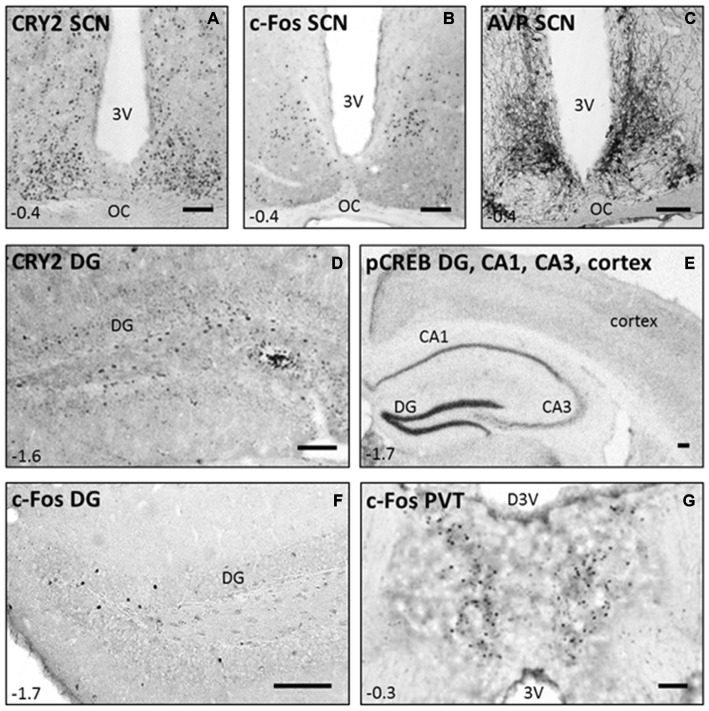
**Immunostainings performed in C57Bl/6 mice after they successfully mastered the 3-arm TPL task.** Representative pictures of CRY2, c-Fos, and AVP immunoreactivity in the suprachiasmatic nucleus (SCN; **A−C**), CRY2 in the DG (**D**, CA3 not shown), pCREB in the DG, CA1, CA3 and cortex **(E)**, and c-Fos in the DG and paraventricular nucleus (PVT; **F,G**). Coordinates relative to bregma are shown in the left lower corner of each panel (according to the mouse brain atlas: Franklin and Paxinos, 1997). Scale bars in the right lower corner of each panel indicate 100 μm in all panels. 3 V = third ventricle; OC = Optic Chiasm; D3 V = dorsal third ventricle.

**Figure 7:**
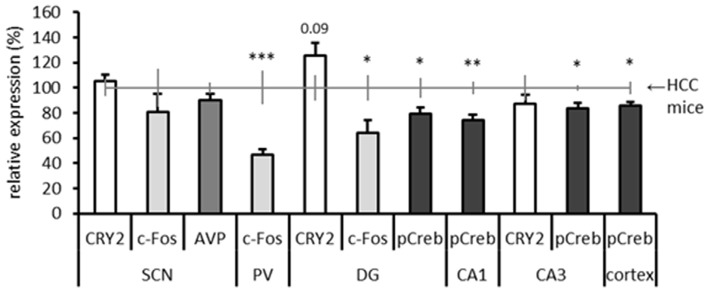
**Summary of results of all performed immunostainings in investigated brain regions.** Expression levels are relative to home cage control (HCC) mice, which are depicted by the horizontal gray line at 100% (including error bars). The Dentate gyrus (DG), Cornu Ammonis areas 1 and 3 (CA1; CA3) are subregions of the hippocampus. Same markers are indicated by same grayscale colors. All error bars represent SEM. Statistical evaluations (two-tailed unpaired *t*-tests) of expression in HCC vs. TPL trained mice are included: *p < 0.05, **p < 0.01, ***p < 0.001. CRY2 expression in the DG showed a statistical trend toward significance (*p* = 0.09).

Representative pictures of the immunostainings of TPL-trained mice are shown in Figure 6. Cry-2 immunoreactivity was found in the nuclei of neurons, mainly located in the SCN and surrounding brain regions (see Figure 6A), and the DG of the hippocampus (see Figure 6D). pCREB-immunoreactivity, also present in neuronal nuclei, was most strongly expressed in the DG of the hippocampus (see Figure 6E), whereas AVP-immunoreactivity present in the cytoplasm of neurons was found most strongly in regions of the hypothalamus, including the SCN as shown previously with this antibody (Van der Zee and Bult, 1995; see Figure 6C). c-Fos-immunoreactivity was present throughout the brain, including the SCN (Figure 6B), the DG of the hippocampus (Figure 6F), and the PVT (Figure 6G).

(Semi)-quantitative results of the different immunostainings are summarized in Figure 7. Because different quantification methods were used, all results are expressed as percentage relative to the HCC group (set at 100% expression) for optimal comparison. In the SCN we analyzed c-Fos, CRY2 and AVP, and in the hippocampus we analyzed CRY2, c-Fos and pCREB in those subregions where specific and clear immunostaining was present. pCREB was also analyzed in the cortex for its function in the storage of long-term memory. C-Fos was additionally analyzed in the (anterior) PVT, because a clear signal (specific staining of neurons) was observed. No differences were found between TPL-trained and HCC mice at the level of the SCN for the investigated markers. This is in line with our earlier finding that the SCN is not essential for TPL (Mulder et al., 2014). Notably, a significant decrease in c-Fos positive cell-counts were found in the PVT and in the DG. The optical density of pCREB-positive cells located in DG, CA1, CA3 and Somatosensory Barrel Cortex was found to be significantly decreased in TPL-trained mice compared to HCC mice. In contrast, CRY2 immunoreactivity showed a 26% increase in the DG of TPL-trained mice compared to HCC mice (Cohen’s *d* = 0.89; effect-size *r* = 0.41), which was at the border of significance (*p* = 0.09).

## Discussion

Here, we studied CD1 mice in the TPL paradigm (first aim of the study) and showed that the CD1 mouse, which is relatively poor in hippocampal functioning and ages relatively fast, cannot master the 3-arm TPL task. They can master the 2-arm TPL task, but middle-aged and notably aged CD1 mice use an ordinal (non-circadian) strategy. This strategy most likely depends on the striatum (they learned a sequence of events) instead of a circadian strategy by which they use their circadian system as a timing device (in the latter case referred to as cTPL). These results further stress the aging-sensitivity of TPL, and are in support of hippocampal involvement in cTPL performance. Thereafter, we set out to identify (parts of) the neuronal substrate underlying successful TPL performance (second aim of the study) by way of IHC analyses of CRY2, c-Fos, AVP and pCREB in brain sections of C57Bl/6 mice that mastered the 3-arm TPL task. The hippocampus showed significant changes for c-Fos and pCREB, further indicating a role of the hippocampus in mastering cTPL. An increased expression of CRY2 by 26% in the DG fits the earlier observation that the 3-arm TPL task is Cry-dependent (Van der Zee et al., 2008). Of interest is the strong change in c-Fos expression in the PVT, a circadian relay station in the thalamus (Moga et al., 1995).

### TPL and Aging

An important step to studying TPL behavior in laboratory settings has been the development of a suitable paradigm, in which animals show consistent cTPL behavior. Recently, we reviewed the long road towards such a functional paradigm (Mulder et al., 2013a). The key has been to find a balanced approach between a reward to motivate animals to choose correct locations (finding food while hungry), in combination with a punishment (response cost) for choosing incorrect locations. Note that a response cost is likely implicit in widespread natural habitats, because traveling to a non-rewarding/predated location will (at best) be costly on energy. Scaling down TPL behavior into a laboratory setting therefore required the artificial implementation of such a response cost, which we have done so by the application of a mild but aversive footshock.

Previously, we investigated TPL for the first time in the context of aging (Mulder et al., 2015). We found that most untrained C57Bl/6 mice were unable to acquire TPL at middle-age (17 months). Surprisingly, some mice did master the task by adapting an alternative (ordinal) TPL strategy. We hypothesize that age-related hippocampal dysfunction, together with age-related circadian system decline caused these untrained mice to adapt this ordinal (non-circadian) TPL strategy, which is presumably less cognitively demanding than cTPL (Mulder et al., 2013a). In contrast, mice trained over their lifespan successfully maintained the circadian strategy (cTPL, learned at young age) until old age (Mulder et al., 2015). At this age however, mice showed signs of behavioral rigidity and a lack to update TOD information. The aging-sensitivity of the TPL paradigm was further stressed by the failure of middle-aged CD1 mice (in contrast to middle-aged C57Bl/6 mice) to master the 3-arm TPL task. It remains to be determined, however, whether young CD1 mice can master this task. An overview of the previously obtained results and the currently obtained results are shown in Table 2.

**Table 2:** **Overview of previous and current findings**.

	C57Bl/6 mice		CD1 mice	
Age	Young/Middle aged	Aged/Old	Young/Middle aged	Aged/Old
2-arm TPL	n.d.	n.d.	**Successful**	**Successful**
			**Use ordinal strategy**	**Use ordinal strategy**
3-arm TPL	*Successful*	*Fail*	**Fail**	**Fail**
	Use circadian strategy	Some use ordinal strategy	**No strategy**	**No strategy**
	**IHC analyses**
Spontaneous alternation scores	*Ca. 70%*	*Ca. 70%*	**Ca. 50%**	**Ca. 50%**
Circadian system	*intact*	*Age deteriorated*	**intact**	**Age deteriorated**
**Cross maze**	**No preference**	**Mainly hippocampal**	**Mainly striatal**	**striatal**

*A summary of the previous findings (in italic; see also Mulder et al., 2015) and the current findings (in bold). Analyses of the neuronal substrate by way of immunohistochemistry (IHC) has been limited to young and middle-aged C57Bl/6 mice that mastered cTPL successfully in the 3-arm TPL set up. n.d. = not determined*.

The striatum and hippocampus are widely held to be components of distinct memory systems that can guide competing behavioral strategies (Berke et al., 2009; Hagewoud et al., 2010). While hippocampus-dependent episodic memory is particularly age sensitive, the striatal system is more age-resistant (Churchill et al., 2003; Nilsson, 2003). We suggest that (c)TPL requires the plasticity of an intact hippocampus (hippocampal-driven strategy), while ordinal TPL, as used by aged mice in general and CD1 mice in particular, may instead rely more on the aging-resistant striatal (procedural; striatal-driven strategy) memory system. This hypothesis may be confirmed in future studies, for instance by selective lesions in the hippocampus and striatum. To what extent the striatum is involved in (c)TPL is currently unknown and requires future experiments.

### The Neuronal Substrate of TPL

Here, we applied IHC on the brains of young to middle-aged C57Bl6/J mice that had successfully mastered cTPL. These mice were sacrificed the day after their last TPL test day on a test-session time point, together with HCC mice. We investigated the expression of vasopressin (AVP, the main circadian output of the SCN), CRY2, and a plasticity marker (pCREB) in the SCN, but we found no differences compared to HCC mice. This corroborates with our SCN lesion results which have indicated that the SCN is not the primary clock used in cTPL (Mulder et al., 2014). The current findings do not support a modulating role of the SCN in TPL. Perhaps other markers should be investigated. Gritton et al. showed that cholinergic signaling from the basal forebrain to the SCN can serve as a temporal timestamp attenuating SCN photic-driven rhythms during cognitive training (Gritton et al., 2013). Cholinergic markers may thus be interesting to further investigate this putative SCN gating mechanism. Noteworthy, in contrast to rats, the SCN in mice does not contain cholinergic neurons, but the SCN of both species do express cholinergic receptors extensively (Van der Zee et al., 1991; Hut and Van der Zee, 2011).

We showed that the most pronounced difference between TPL trained and HCC mice was found in c-Fos expression in the PVT, which has been referred to as a circadian system relay station (Moga et al., 1995). The PVT receives input from all major components of the circadian timing system, including the SCN, subparaventricular zone, the intergeniculate leaflet, and the retina. In addition, the PVT is connected to brain areas involved in learning and memory, including the ventral striatum, amygdala, entorhinal cortex, hippocampus, and cortex (Pickard, 1982; Watts et al., 1987; Moga et al., 1995). The PVT may thus be an interesting target area for future lesion studies in the context of cTPL.

Notably, a significant decrease in c-Fos positive cell-count in the DG and the optical density of pCREB-positive cells located in DG, CA1, CA3 and Somatosensory Barrel Cortex was found in TPL trained mice compared to HCC mice. One explanation for these decreased expression levels is that mice were extensively trained. It has been shown that, with extensive training, c-Fos is attenuated in most brain regions (Bertaina-Anglade et al., 2000). Moreover, c-Fos and pCREB are related as pCREB stimulates the expression of c-Fos (Sheng and Greenberg, 1990). In extensively trained animals, the hippocampus may be devoted to the learned task (retention rather than acquisition) activating only the cells devoted to this task. From another perspective, training may increase synchronization of hippocampal neurons, causing less cells to be active at one given time point. It would therefore be interesting to also investigate these markers during the learning (acquisition) phase of TPL. Kononen et al. (1990) showed that in the rat brain, c-Fos levels show a circadian rhythmicity, with peak expression in the active (dark) phase. Therefore, another explanation for the decreased expression levels may be that TPL testing induced a phase shift (advance) in c-Fos (and pCREB) circadian expression relative to the “normal” expression pattern in HCC mice.

The 26% upregulation of CRY2 in the DG of TPL-trained mice compared to HCC mice is an interesting finding. cTPL likely involves the hippocampus, which is known to be involved in spatial navigation and episodic and episodic-like memory. The DG is one of the few brain areas where adult neurogenesis occurs, and thought to be particularly involved in the formation of new episodic memories (Amaral et al., 2007; Treves et al., 2008). It has been proposed that experience-related cues (cognitive training) may act as a zeitgeber to the hippocampus, where local timekeeping mechanisms may be entrained (Gritton et al., 2013). Whether *Cry*, but not *Per* genes are essential for temporal coding in the hippocampus remains to be further investigated, for example by using hippocampus specific *Cry* and *Per* knockout mice.

### TPL as a Model for Episodic-Like Memory

Numerous clinical studies have established a direct correlation between abnormal circadian clock functions and the severity of neurodegenerative disorders, suggesting a functional link between the circadian clock and age-associated decline of brain functions (Kondratova and Kondratov, 2012). cTPL demonstrates that animals can form so-called “tripartite memory codes” consisting of associated what, where, and when information, resembling the content of human episodic memory. This type of hippocampus-dependent memory is particularly susceptible to the pathologies of aging and neurodegenerative disease (Squire et al., 2004; Stranahan et al., 2008; Berke et al., 2009). Therefore, TPL may have specific potential as an animal model for episodic memory and aging. It has been demonstrated that rats and mice are able to associate object-, spatial-, and temporal information after a single exposure to such stimulus constellations (Dere et al., 2005a,b, 2007). Hence, the first trials of cTPL training are potentially episodic-like in nature, and link cTPL to episodic-like memory. Behavioral models based on temporal information are scarce, yet essential to test interventions that potentially improve detrimental effects of aging and (episodic) memory related diseases like Alzheimer’s disease (AD; Dere et al., 2005a,b). Indeed, patients suffering from AD are often said to be disorientated in time and place, and memories of when and where things happened (episodic memory) are among the first to be affected in AD patients. Aging is characterized by cognitive decline (Winocur, 1992; Nilsson, 2003; Hedden and Gabrieli, 2004; Burke and Barnes, 2006), as well as circadian system deterioration (Turek et al., 1995; Van der Zee et al., 1999; Hofman and Swaab, 2006; Brown et al., 2011; Kondratova and Kondratov, 2012). We therefore predicted that cTPL is specifically age sensitive, as shown in this study and earlier work (Mulder et al., 2015).

Whether and to what extent the (c)TPL task is an episodic-like type of memory paradigm remains a matter of debate. Taken the seven criteria of Pause et al. (2013), it does not fulfill the criteria as rehearsal is a critical aspect of TPL. Nevertheless, the task requires integrated what–where–when components, 24 h-retention, relies on the hippocampus and is sensitive to aging. Moreover cTPL requires specific knowledge of TOD rather than a discrimination of relative recency, as in most other episodic memory paradigms. For these reasons (c)TPL can significantly contribute to our understanding of mechanisms underlying episodic-like memory or specific temporal aspects of episodic-like memory. The TPL task could be viewed as a collection of multiple episodic memories, and/or a semantic memory task using specific episodic information. Moreover, it may shed light on the way TOD information is encoded into memory. Similarly humans can often remember the TOD of specific events within the range of some hours (a significant event happened in the early or late morning, for example). Interestingly, also in cTPL mice remember the TOD within a range of approximately 1.5 h (Mulder et al., 2013a, 2015). The existence of circadian-timed episodic-like memory has also been claimed in other species, such as bees (Pahl et al., 2007). Taken together, TPL and particularly cTPL can functionally be linked to episodic-like memory even if the task seems more related to semantic memory due to the repeated trials needed to successfully master the task. We suspect that TPL depends on episodic memory, but due to its functional nature, also entail the translation of experienced episodes into semantic rules acquired by training. A next step would be to directly compare underlying neuronal substrates in an established episodic-like memory task and cTPL.

## Conflict of Interest Statement

The authors declare that the research was conducted in the absence of any commercial or financial relationships that could be construed as a potential conflict of interest.
